# An investigation into the conversion of In_2_O_3 _into InN nanowires

**DOI:** 10.1186/1556-276X-6-311

**Published:** 2011-04-07

**Authors:** Polina Papageorgiou, Matthew Zervos, Andreas Othonos

**Affiliations:** 1 Department of Physics, Research Centre of Ultrafast Science, University of Cyprus, P.O. Box 20537, Nicosia, 1678, Cyprus; 2 Nanostructured Materials and Devices Laboratory, Department of Mechanical Engineering, Materials Science Group, School of Engineering, University of Cyprus, P.O. Box 20537, Nicosia, 1678, Cyprus

## Abstract

Straight In_2_O_3 _nanowires (NWs) with diameters of 50 nm and lengths ≥2 μm have been grown on Si(001) via the wet oxidation of In at 850°C using Au as a catalyst. These exhibited clear peaks in the X-ray diffraction corresponding to the body centred cubic crystal structure of In_2_O_3 _while the photoluminescence (PL) spectrum at 300 K consisted of two broad peaks, centred around 400 and 550 nm. The post-growth nitridation of In_2_O_3 _NWs was systematically investigated by varying the nitridation temperature between 500 and 900°C, flow of NH_3 _and nitridation times between 1 and 6 h. The NWs are eliminated above 600°C while long nitridation times at 500 and 600°C did not result into the efficient conversion of In_2_O_3 _to InN. We find that the nitridation of In_2_O_3 _is effective by using NH_3 _and H_2 _or a two-step temperature nitridation process using just NH_3 _and slower ramp rates. We discuss the nitridation mechanism and its effect on the PL.

## Introduction

Group III-Nitride (III-N) semiconductors have been investigated extensively over the past decades due to their applications as electronic and optoelectronic devices. In addition, they are promising for the realization of high efficiency, multi-junction solar cells since their band-gaps vary from 0.7 eV in InN through to 3.4 eV in GaN up to 6.2 eV in AlN; thereby, allowing the band gaps of the ternaries In*_x_*Ga_1-*x*_N and Al*_x_*Ga_1-*x*_N to be tailored in between by varying *x*. Nanowires solar cells (NWSCs) are also receiving increasing attention but so far they have been fabricated from Si and metal-oxide (MO) NWs. Nitride NWs such as InN [1], GaN [2] and AlN [3] are, therefore, promising for the realization of full-spectrum third generation NWSCs. However, their growth and properties must be understood beforehand in order to make nanoscale devices. So far we have grown InN [1] and GaN NWs [2] using the direct reaction of In or Ga with NH_3_, while more recently we showed that Ga_2_O_3 _NWs may be converted to GaN by post-growth nitridation using NH_3 _and H_2 _[4]. Here, we have undertaken a systematic investigation into the conversion of In_2_O_3 _to InN NWs, which has not been carried out previously by others, thereby complementing our earlier work on the conversion of Ga_2_O_3 _to GaN NWs.

Therefore, we have grown straight In_2_O_3 _NWs with diameters of 50 nm and a high yield and uniformity. We find that the post-growth nitridation of In_2_O_3 _NWs using NH_3 _leads to the elimination of the NWs above 600°C. The In_2_O_3 _NWs are preserved for temperatures less than 700°C but are not converted into InN even after long nitridation times of 6 h. However, the nitridation process was enhanced significantly via the use of H_2 _or by employing a two-step temperature nitridation process, which also lead to a suppression of the photoluminescence (PL) peak at 550 nm similar to the nitridation of Ga_2_O_3 _NWs [4].

## Experimental method

Initially In_2_O_3 _NWs were grown using an atmospheric pressure chemical vapour deposition (APCVD) reactor described elsewhere [5]. For the growth of In_2_O_3 _NWs, 0.2 g of fine In powder (Aldrich, Cyprus, Mesh 100, 99.99%) was weighed and loaded in a quartz boat, while square pieces of *n*^+ ^Si(001) ≈ 7 mm × 7 mm, coated with ≈1.0 nm of Au, were loaded at various distances from the In. The Au layer was deposited via sputtering using Ar under a pressure of ≈10^-2 ^mBar. The boat was positioned directly above the thermocouple used to measure the heater temperature at the centre of the 1" quartz tube (QT). Another quartz boat with ≈5 ml of de-ionised (DI) H_2_O was positioned at the inlet of the tube. After loading the boats at room temperature (RT), Ar (99.999%) was introduced at a flow rate of 500 standard cubic centimetres per minute (sccm) for 10 min. Following this, the temperature was ramped to 850°C under a flow of 50 sccm Ar using a ramp rate of 30°C/min. Upon reaching the growth temperature (*T*_G_), the flow of Ar was maintained at 50 sccm for 30 min in order to grow the In_2_O_3 _NWs after which the reactor was allowed to cool down in a flow of 50 sccm of Ar for at least 30 min. The sample was always removed only when the temperature was lower than 100°C.

The nitridation of the In_2_O_3 _NWs was carried out in a new 1" QT without any solid precursors. After loading each sample with In_2_O_3 _NWs from the downstream side, a flow of 500 sccm Ar was introduced for 10 min after which the temperature was ramped to the nitridation temperature (*T*_N_) under a flow of NH_3 _that varied between 125 and 250 sccm using a ramp rate of 30°C/min. Upon reaching *T*_N_, the same flow of NH_3 _was maintained for various times between 1 and 6 h after which the reactor was allowed to cool down to RT under the same flow of NH_3_. A list of the different temperatures, nitridation times and NH_3 _gas flows used for the nitridation of the In_2_O_3 _NWs are shown in Table 1. Similarly nitridation was carried out using NH_3 _and H_2_. In this case, the temperature was ramped to 500°C under a flow of NH_3 _and H_2 _whose relative flows varied using a ramp rate of 30°C/min. Upon reaching *T*_N_, the same flow of NH_3 _and H_2 _was maintained for 1 h. The total flow of NH_3 _and H_2 _was kept constant at 200 sccm and a list of the different flows of H_2 _is listed in Table 1. Finally, we carried out a two-step temperature process. In this case, the temperature was ramped to 500°C under 125 sccm of NH_3 _using a ramp rate of 10°C/min. Upon reaching *T*_N_, the same flow of NH_3 _was maintained for 1 h. Then, the temperature was ramped to 700°C and the same flow of NH_3 _was maintained for 30 min after which the reactor was allowed to cool down to RT.

**Table 1:** Summary of post-growth nitridation conditions for the conversion of In_2_O_3 _NWs to InN.

(I) *T*_N _(°C)		(II) *t *(h)		(III) %H_2_	
CVD797	500°C	CVD850	500°C, 3 h	CVD855	10
CVD788	600°C	CVD853	500°C, 6 h	CVD856	20
CVD790	800°C	CVD795	600°C, 1 h	CVD857	40
CVD791	900°C	CVD849	600°C, 2 h	CVD859	80
		CVD848	600°C, 3 h		

Initially a flow of 500 sccm of Ar was introduced into the reactor after which the temperature was ramped to *T*_N _at 30°C/min under a flow of (I) 250 sccm of NH_3_, (II) 125 scmms of NH_3 _and (III) under different flows of NH_3 _and H_2_, but keeping the total flow constant at 200 sccm. Upon reaching *T*_N_, the same flows were maintained for 1 h at various temperatures (I), different nitridation times at 500 and 600°C (II) and for 1 h at 500°C (III).

The morphology of the as grown In_2_O_3 _NWs and those treated with NH_3 _were examined with a TESCAN scanning electron microscope (SEM), while their crystal structure and phase purity were investigated using a SHIMADZU, X-ray diffraction (XRD-6000), with Cu-Ka source, by performing a scan of θ - 2θ in the range between 10° and 80°. Finally, PL measurements were carried using above bandgap (approx. 3.75 eV [6]) excitation at 267 nm. The pulse excitation was the second harmonic of a beam from an *optical parametric amplifier *pumped with a mode-locked TiSapphire laser. The pulses were 100 fs FWHM at a repetition rate of 250 kHz. The energy per pulse incident on the samples was 40 pJ over a spot of 2 mm in diameter.

## Results and discussion

**Figure 1:**
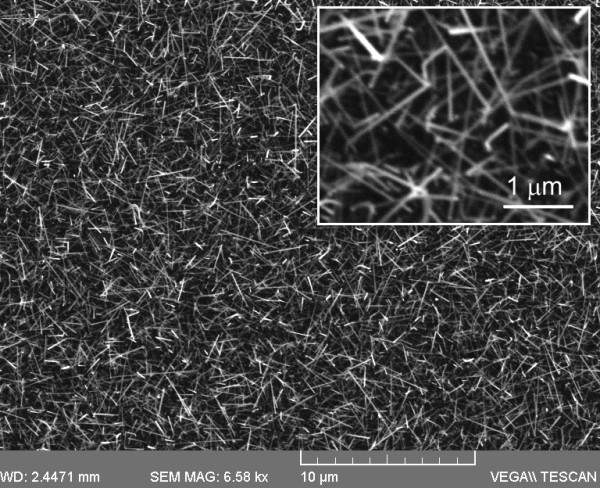
**Typical SEM image of In_2_O_3 _NWs obtained on 1.1 nm Au/Si(001)**.

**Figure 2:**
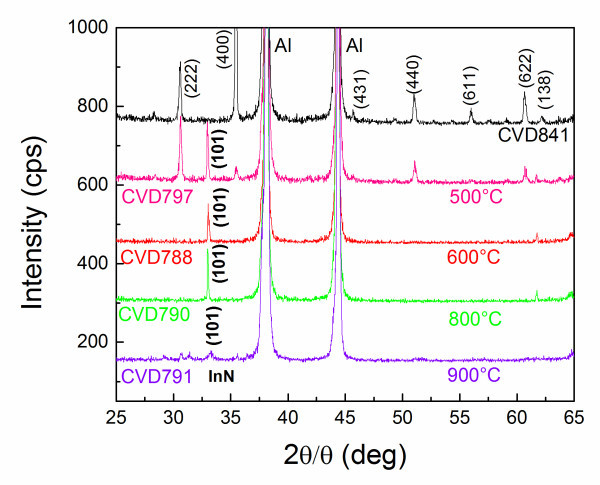
**XRD of In_2_O_3 _NWs obtained after nitridation at different temperature as listed in Table 1**. Note that CVD841 shown at the top corresponds to the as grown In_2_O_3 _NWs. The InN related peaks are shown in bold, while the Al peaks belong to the holder and have also been identified.

Previously, we obtained In_2_O_3 _NWs by dry oxidation at 700°C [7]. A high yield of In_2_O_3 _NWs with an average diameter of ≈100 nm and lengths of ≈1 μm was obtained on Si(111) and quartz. However, these In_2_O_3 _NWs were slightly tapered; their diameters were larger and lengths were shorter compared to the In_2_O_3 _NWs obtained here by wet oxidation. Moreover, the distribution of the In_2_O_3 _NWs obtained by wet oxidation was far superior and much more uniform compared to those obtained by dry oxidation. A typical image of In_2_O_3 _NWs that were obtained at *T*_G _= 850°C by wet oxidation is shown in Figure 1. It should be pointed out that a high yield and uniform distribution of In_2_O_3 _NWs extending over 1 cm^2 ^was obtained when the distance between the In and the Au/n^+^Si (001) was ≥15 mm, which led to a light blue-like deposit. The In_2_O_3 _NWs have diameters of ≈50 nm, lengths ≥2 μm and exhibited clear peaks in the XRD as shown in Figure 2 by the top curve, corresponding to the body centred cubic (bcc) crystal structure of In_2_O_3 _with *a *= 10.12 Å, in agreement with Dai et al. who obtained twisted In_2_O_3 _NWs by wet oxidation [8]. The In_2_O_3 _NWs shown in Figure 1 are straight [9,10] and in our case In_2_O_3 _NWs grow by a simple chemical route involving the following reaction: 2In + 3H_2_O → In_2_O_3 _+ 3H_2 _[8]. Wet oxidation is a facile method and generally occurs faster than dry oxidation. No NWs were obtained on plain Si(001), suggesting the growth of In_2_O_3 _NWs occurs via the vapour-liquid-solid (VLS) mechanism with Au acting as the catalyst. In this case, Au NPs absorb In until they become supersaturated after which In_2_O_3 _NW growth commences via the reaction of In with H_2_O as outlined above.

**Figure 3:**
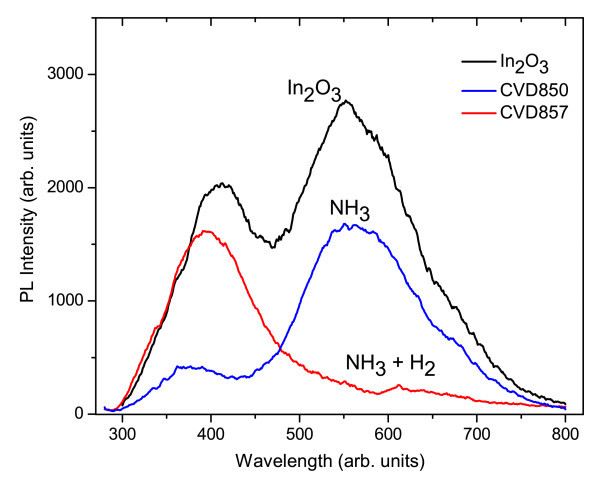
**PL spectrum of In_2_O_3 _NWs as grown and after nitridation using NH_3 _only or NH_3 _and H_2_**.

The PL spectrum following excitation at 267 nm at 300 K consisted of two broad peaks, centred at 400 and 550 nm as shown in Figure 3 Similar peaks in the PL have been observed by Yan et al. [11] who obtained a broad luminescence band centred at 395 nm from In_2_O_3 _nanorods, Liang et al. [12] who found a peak at 470 nm from In_2_O_3 _nanofibres and Wu et al. [13] who observed two distinct peaks at 416 and 435 nm from In_2_O_3 _nanowires. It is important to point out that these peaks are commonly attributed to the presence of oxygen vacancies.

Next, we will describe the conversion of In_2_O_3 _NWs into InN and in particular consider the nitridation of In_2_O_3 _NWs at different temperatures. To begin with In_2_O_3 _NWs were subjected to 250 sccm of NH_3 _for 1 h at various temperatures between 500 and 900°C as listed in Table 1.

The XRD spectra of the In_2_O_3 _NWs treated at different temperatures is shown in Figure 2. As can be seen most of the oxide peaks disappear at temperatures >600°C. However, a new peak appears, which corresponds to the (101) crystallographic direction of InN [1]. Furthermore, SEM images reveal that the In_2_O_3 _NWs have been eliminated above 600°C, but a thin layer of InN remains on the Si(001). Evidently, the nitridation of the In_2_O_3 _NWs is destructive above 600°C due to the fast decomposition of In_2_O_3 _to In_2_O, which is a gas. We should also point out that in addition to the temperature we also varied the nitridation time. In particular, we carried out nitridations of In_2_O_3 _NWs at 500 and 600°C under a flow of 125 sccm NH_3 _for different times as described in Table 1.

**Figure 4:**
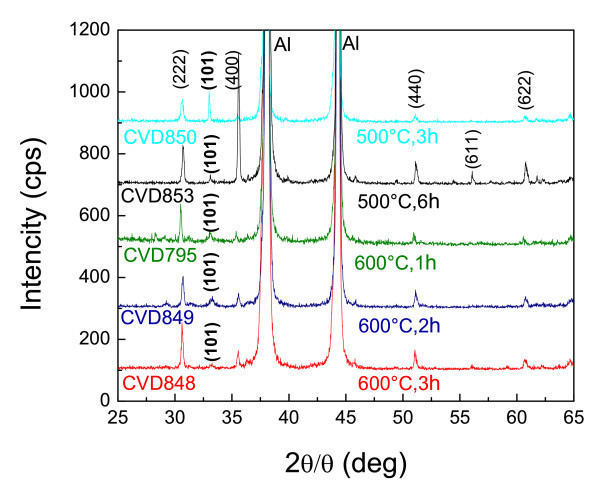
**XRD of In_2_O_3 _NWs obtained after nitridation at 500 and 600°C for different times as described in Table 1**.

**Figure 5:**
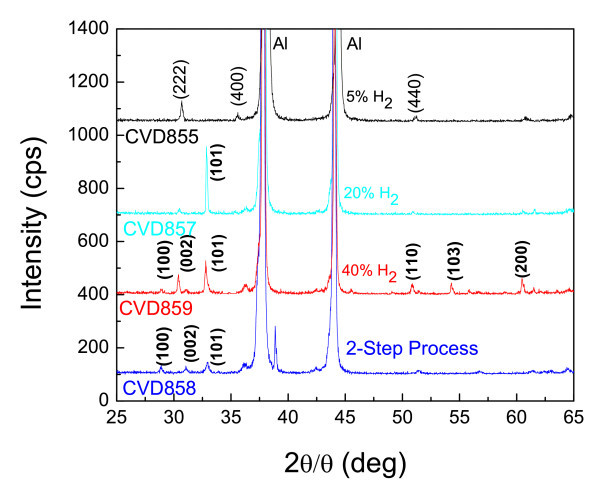
**XRD of In_2_O_3 _NWs obtained after nitridation at 500°C under various flows of NH_3 _and H_2 _as described in Table 1**. The curve at the bottom corresponds to the two-step temperature nitridation process.

Again the conversion of In_2_O_3 _NWs to InN appears to be incomplete as can be clearly seen from the XRD spectra in Figure 4 where one can observe the presence of In_2_O_3 _peaks and just one peak at (101) corresponding to InN. In order to achieve the efficient conversion of In_2_O_3 _NWs to InN without eliminating them, we used two different approaches. In the first one, we have carried out post-growth nitridation, which included H_2 _as shown in Table 1 and in the second approach, we have utilised a two-step temperature nitridation process. The corresponding XRD spectra are shown in Figure 5. As can be seen from the XRD spectra, H_2 _plays a significant role in the removal of the oxygen and thus all major oxide peaks are eliminated and the conversion to InN is achieved with 40% H_2_. As already described above, NH_3 _alone does not promote the efficient conversion of In_2_O_3 _NWs into InN at temperatures between 500 and 600°C. This is likely due to the formation of an InN shell around the In_2_O_3_, which prevents the diffusion of N into the In_2_O_3 _core. However, H_2 _appears to promote the conversion of In_2_O_3_into InN [14].

In addition, the two-step process lead to the effective conversion of In_2_O_3 _NWs to InN using just NH_3_. In this case, the temperature was ramped at 10°C/min up to 500°C and held constant over a period of 1 h, after which the temperature was ramped again slowly to 700°C in order to promote the nitridation. Recall that the In_2_O_3 _NWs were eliminated during a single-step nitridation process at 700°C using a fast ramp rate of 30°C/min. However, it should be noted that the NWs treated by this two-step temperature nitridation process were bent probably due to the fact that the crystal structure changes from bcc to the hexagonal wurtzite structure, and there is a non-uniform strain distribution between the core and shell. The effect of the post-growth nitridations on the PL of the In_2_O_3 _NWs is shown in Figure 3.

In the case of the nitridation using just NH_3 _for 3 h at 500°C, one may observe that there is no substantial change in the shape of the PL of the In_2_O_3 _NWs except from the fact that the PL intensity has been reduced. However, the nitridation of the In_2_O_3 _NWs using NH_3 _and H_2 _leads to a clear suppression of the peak at 550 nm, which is attributed to oxygen consistent with previous investigations on Ga_2_O_3 _[4]. The peak around 400 nm maybe attributed to In vacancies [15], but not O_2 _as commonly suggested [11-13]. However, further work is required to clarify the origin of the PL peak around 400 nm.

## Conclusions

Straight In_2_O_3 _NWs with diameters of 50 nm, lengths ≥2 μm and a bcc crystal structure have been grown on Au/Si(001) via the wet oxidation of In at 850°C. These exhibited two broad peaks in the PL, centred around 400 and 550 nm. The post-growth nitridation of In_2_O_3 _NWs was found to be effective by using NH_3 _and H_2 _at 500 and 600°C or a two-step temperature, nitridation process at 500 and 700°C. This lead to a suppression of the PL peak around 550 nm related to O_2 _consistent with previous investigations on Ga_2_O_3_. In contrast, single-step temperature, nitridations using just NH_3_, carried out with fast ramp rates above 600°C lead to the complete elimination of the In_2_O_3 _NWs, while they were not effective at 500 and 600°C.

## Abbreviations

APCVD: atmospheric pressure chemical vapour deposition; bcc: body centred cubic; DI: de-ionised; MO: metal-oxide; NWs: nanowires; NWSCs: nanowires solar cells; PL: photoluminescence; QT: quartz tube; RT: room temperature; SEM: scanning electron microscope; VLS: vapour-liquid-solid; XRD: X-ray diffraction.

## Competing interests

The authors declare that they have no competing interests.

## Authors' contributions section

MZ and PP carried out the growth, scanning electron microscopy and x-ray diffraction measurements. AO carried optical characterization. All authors read and approved the final manuscript.
